# Latent Space Representation of Human Movement: Assessing the Effects of Fatigue

**DOI:** 10.3390/s24237775

**Published:** 2024-12-04

**Authors:** Thomas Rousseau, Gentiane Venture, Vincent Hernandez

**Affiliations:** 1 Faculty of Odontology, University of Reims Champagne-Ardenne, 51100 Reims, France; thomas.rousseau@etudiant.univ-reims.fr; 2 Department of Mechanical Engineering, The University of Tokyo, Tokyo 113-8654, Japan; venture@g.ecc.u-tokyo.ac.jp

**Keywords:** fatigue, human activity recognition, deep learning, adversarial autoencoder, inertial measurement unit, ground reaction force

## Abstract

Fatigue plays a critical role in sports science, significantly affecting recovery, training effectiveness, and overall athletic performance. Understanding and predicting fatigue is essential to optimize training, prevent overtraining, and minimize the risk of injuries. The aim of this study is to leverage Human Activity Recognition (HAR) through deep learning methods for dimensionality reduction. The use of Adversarial AutoEncoders (AAEs) is explored to assess and visualize fatigue in a two-dimensional latent space, focusing on both semi-supervised and conditional approaches. By transforming complex time-series data into this latent space, the objective is to evaluate motor changes associated with fatigue within the participants’ motor control by analyzing shifts in the distribution of data points and providing a visual representation of these effects. It is hypothesized that increased fatigue will cause significant changes in point distribution, which will be analyzed using clustering techniques to identify fatigue-related patterns. The data were collected using a Wii Balance Board and three Inertial Measurement Units, which were placed on the hip and both forearms (distal part, close to the wrist) to capture dynamic and kinematic information. The participants followed a fatigue-inducing protocol that involved repeating sets of 10 repetitions of four different exercises (Squat, Right Lunge, Left Lunge, and Plank Jump) until exhaustion. Our findings indicate that the AAE models are effective in reducing data dimensionality, allowing for the visualization of fatigue’s impact within a 2D latent space. The latent space representation provides insights into motor control variations, revealing patterns that can be used to monitor fatigue levels and optimize training or rehabilitation programs.

## 1. Introduction

Fatigue is an important factor in sports science that can significantly influence recovery, training effectiveness, athletic performance, and overall athlete well-being. Understanding and predicting fatigue is essential to optimize recovery protocols and training plans, ultimately improving performance outcomes. Monitoring fatigue also plays a crucial role in preventing overtraining and reducing the risk of injuries. Fatigue states reflect the body’s rate of adjustment to changes during demanding tasks, such as physical exercise [1], and highlight the dynamic interaction between physiological systems and their adaptation over time [2]. Given its multifactorial nature, the assessment of fatigue involves both qualitative and quantitative approaches [3,4].

Qualitative methods to assess fatigue mainly involve subjective evaluations through surveys and questionnaires. Common tools include the Recovery Stress Questionnaire (REST-Q) [5] and the Profile of Mood States (POMS) [6]. These questionnaires are widely used by coaches due to their ease of implementation [7]. They help capture an individual’s perceived state of fatigue, covering aspects such as stress levels and recovery quality [8]. However, the results can be biased by variations in individual effort perception, which are influenced by psychological, physiological, and experiential factors [9].

While qualitative assessments are useful, they are often complemented by quantitative measurements that directly capture physiological and biomechanical markers. Methods like surface electromyography (sEMG) provide valuable insight into muscle activation patterns [10], but require controlled environments and time-consuming data processing. Another approach involves using force plates to measure the Ground Reaction Force (GRF) and variations in the Center of Pressure (CoP). GRF provides insights into the mechanical interaction between the body and the ground [11], allowing the assessment of movement patterns, injury risk [12], and performance optimization [13]. Fatigue-induced changes, such as increased muscle activation variance, can be detected by analyzing the CoP and GRF [14,15,16].

Low-cost alternatives to traditional force plates include the Wii Balance Board (WiiBB), which provides a reliable solution to estimate the Center of Pressure (CoP) variation through its four sensors [17]. The WiiBB has been validated for postural assessment in both young [18] and elderly populations [19]. Another alternative sensor for fatigue assessment is the Inertial Measurement Unit (IMU), which integrates accelerometers and gyroscopes. IMUs are widely used in biomechanics for rehabilitation and performance enhancement [20], providing valuable information on human physical activity [21,22], particularly in relation to fatigue-induced changes during physical tasks [23,24].

Human Activity Recognition (HAR) is a specialized research area that combines machine learning and sensor data to analyze and monitor human movements [25,26]. Using multimodal sensor data, HAR can offer real-time feedback and personalized monitoring, which is particularly valuable for fatigue assessment. Despite its advantages, HAR often focuses on classification tasks [27], which, while informative, may not capture the nuanced changes in motor control associated with fatigue.

Recent advances in HAR [27] have enabled multimodal sensor analysis [28], with a primary focus on classification tasks [29]. Although these classification techniques provide valuable information, they are often limited in offering a comprehensive understanding of activity and fatigue. Therefore, to effectively interpret the complexities of fatigue, it is essential to move beyond classification and closely examine the nuances of changes in motor control, thus fully capturing and understanding the underlying dynamics of fatigue. Several studies have shown that data visualization improves decision-making processes for both experts and non-experts [30,31,32]. Effective visualization techniques, especially those based on dimensionality reduction, are important for understanding the underlying patterns and features of high-dimensional data. By transforming complex data into more interpretable formats, dimensionality reduction not only facilitates the interpretation of fatigue patterns and interactions, but also makes these insights more accessible to non-professionals. Thus, effective data visualization plays a crucial role in both understanding fatigue and communicating complex findings more effectively.

Given the high dimensionality and complexity of sensor data, effective data visualization through dimensionality reduction is crucial. These techniques not only facilitate the interpretation of complex data, but also improve decision-making processes for experts and non-experts [30,31]. By transforming high-dimensional sensor data into interpretable formats, dimensionality reduction would provide clearer information on activities and fatigue patterns [33,34].

This study uses data from Wii Balance Boards and Inertial Measurement Units (IMUs) to compare the effectiveness of different sensor combinations in assessing fatigue. Adversarial AutoEncoders (AAEs) are employed for dimensionality reduction of sensor data, enabling the visualization of fatigue’s impact in a two-dimensional latent space. The focus is on understanding how fatigue affects motor control by analyzing changes in the distribution of data points within this latent space. The goal is to assess motor changes associated with fatigue and provide a visual representation of these effects. It is hypothesized that increased fatigue will cause significant changes in point distribution, which will be analyzed using clustering techniques to identify fatigue-related patterns.

## 2. Methods

For this study, a group of 30 healthy male participants was recruited. Eligible participants were healthy adult men between 20 and 30 years of age with no current injuries or disabilities affecting upper or lower limb function. Only physically active individuals capable of safely performing the prescribed exercises were included. Participants with medical conditions that could interfere with participation or affect physical task performance were excluded from the study.

The participants were divided into two groups: Group A (*n* = 20; age: 24.1 ± 2.9 years, height: 173.9 ± 7 cm, weight: 66.6 ± 9.1 kg) and Group B (*n* = 10; age: 23.4 ± 2.5 years, height: 175.5 ± 7 cm, weight: 68.2 ± 13.3 kg). The data from Group A were used to train the initial machine learning models, ensuring that the model could capture a wide variability of motion across different activities. The data from Group B were then used to fine-tune the models and perform fatigue analysis, providing additional information to evaluate the models’ ability to detect fatigue-related changes in motor control.

The experiment was approved by the local ethics committee of the University of Tokyo. All participants provided their written consent in accordance with the Declaration of Helsinki on Human Experimentation.

### 2.1. Experimental Protocol

For this experiment, four different lower body fitness exercises were considered: “Squat”, “Lunge Left”, “Lunge Right”, and “Plank Jump-in”. All exercises were performed in sets of 10 repetitions. Group A performed a set of 10 repetitions for each exercise. Group B performed sets of 10 repetitions for each exercise repeatedly until they reached complete fatigue. Between individual repetitions, participants took 1 to 2 s of rest, 15 to 20 s of rest between sets (only Group B) and a 5 min rest period between different exercises. To minimize bias due to fatigue, the order of the exercises was randomized for both groups. From this point on, only Group B will be used and fatigue analysis will be performed.

WiiBB (Nintendo Co. Ltd, Kyoto, Japan) and IMU sensor (Movella Inc., Henderson, NV, USA) data were collected at 30 and 60 Hz, respectively. Three IMU sensors were used: one on the hip and the other two on each distal part of the forearms to mimic the presence of a smartwatch.

Each repetition of each exercise was segmented and interpolated to 192 frames using cubic spline interpolation. To ensure precise segmentation, synchronized video footage was also collected. The boundaries of each repetition were detected using accelerometer data to detect periods of movement and stillness that were then fine-tuned manually with the video data to ensure precision.

### 2.2. Database

The total GRF and CoP were retrieved from WiiBB sensors [33]. The x-axis of the CoP represents the anteroposterior axis, while the y-axis represents the mediolateral axis. To remove bias due to the initial position for each repetition, the initial Center of Pressure of the mediolateral (X) and anteroposterior (Y) axes was subtracted and the GRF was normalized with the mass of the subject m.

Before each exercise, a static calibration was conducted using a T-pose to perform inertial to segment (I2S) calibration and correct any misalignment of the IMU sensors that may have occurred during previous activities. During the T-pose, it was assumed that all body segments were aligned with the world reference frame, allowing the sensor data to be adjusted accordingly. The orientation of each IMU sensor in relation to the inertial reference frame was used to determine how it should be aligned with the segments of the body. Then, gravity was removed from the accelerometer signals and the acceleration was normalized according to the participant’s height h.

For each repetition, data from all sensors were gathered in a matrix $X_{l}$:

(1) $$X_{l}=\left[\begin{array}{ccc} f_{1}^{\left(1\right)} & \ldots & f_{n}^{\left(1\right)}\\ f_{1}^{\left(2\right)} & \ldots & f_{n}^{\left(2\right)}\\ \ldots & \ldots & \ldots \\ f_{1}^{\left(m\right)} & \ldots & f_{n}^{\left(m\right)} \end{array}\right]\in R^{m\times n}$$

with *m* = 196 representing the number of frames and n the number of time-series variables (Table 1).

The data from the Wii Balance Board and the IMU were filtered using a low-pass filter with a cutoff frequency of 15 Hz and a filter order of 4 to remove high-frequency noise, ensuring that only the relevant lower-frequency components associated with human movement were preserved [35].

Then, a dataset $D_{s}$ is created for each participant with R as the total number of movements and the corresponding output label $y_{l}$ of $X_{l}$ represented as a binary one-hot vector, as follows:

(2) $$D_{s}={\left\{X_{l},y_{l}\right\}}_{l=1}^{R}$$

### 2.3. Standardization

The input data were standardized according to their type (GRF, CoP, acceleration, or angular velocity). To do so, the global mean (μ) and the standard deviation (σ) for each type of data were calculated from the training data from our datasets from Group A for each fold [33]. These statistics were then used to standardize both datasets, ensuring consistency across them as follows:

(3) $$z_{i}=\frac{x_{i}-\mu }{\sigma }$$

### 2.4. AutoEncoder

An AutoEncoder (AE) is a type of artificial neural network used for unsupervised learning, where label information is not provided. Its goal is to compress the input data into a lower-dimensional latent space and then reconstruct the original data, thereby learning a new representation of the input. An AE is composed of two main parts connected by a latent space z. The encoder (4) compresses the input data to z. It maps the input to the latent space through several layers of neurons, where each layer applies a non-linear transformation to the input. The decoder (5) reconstructs the input data from z at its output as closely as possible. This structure allows the AE to effectively reduce the dimensionality of the data while preserving its essential features.

(4) $$f_{\mathrm{Encoder}}:X\in R^{m\times n}\to z\in R^{d}$$

(5) $$f_{\mathrm{Decoder}}:z\in R^{d}\to X^{\prime }\in R^{f\times n}$$

The encoder output layer is designed with 2 neurons, creating a two-dimensional latent space ($z\in R^{2}$), allowing the critical feature of the input to be condensed into a lower-dimensional space. The data distribution is then embedded within this latent space. In order to obtain a continuous and structured latent space, regularization is applied to shape it according to a specific prior distribution. We are applying this regularization through an Adversarial AutoEncoder approach [36].

### 2.5. Adversarial Autoencoder

An Adversarial AutoEncoder (AAE) combines the principles of AE with the adversarial training mechanism introduced in Generative Adversarial Networks [37]. This integration constrains the encoding distribution q(z|X) to match a desired prior distribution p(z), resulting in an aggregated latent space distribution q(z) that aligns with p(z). This alignment allows for a flexible approach to both unsupervised and semi-supervised clustering. The discriminator (7) operates in the latent space z. The structure is defined as follows:

(6) $$f_{\mathrm{AutoEncoder}}:X\in R^{m}\to z\in R^{d}\to X^{\prime }\in R^{m}$$

(7) $$f_{\mathrm{Discriminator}}:z\in R^{d}\to w\in R^{1}$$

(8) $$f_{\mathrm{Adversarial}\;\mathrm{Network}}:X\in R^{m}\to z\in R^{d}\to w\in R^{1}$$

In summary, an AAE is composed of two main components: an AutoEncoder and an Adversarial Network, with the encoder being shared between them. Meanwhile, the Adversarial Network, which includes a discriminator, works to refine this latent space. The discriminator’s role is to distinguish between the true data distribution and the distribution generated by the encoder. During training, the encoder adjusts to fool the discriminator, effectively improving the quality of the latent representations by making them more similar to the true data distribution. This adversarial process improves the encoder’s ability to capture meaningful features in the latent space.

In this study, we used two different AAE models with semi-supervised training. The first model, abbreviated as Res-SSAAE, incorporates basic residual blocks in both the encoder and the decoder [38]. These residual connections help mitigate the vanishing gradient problem and facilitate the training of deeper models to improve the overall performance. Furthermore, inspired by the conditional Generative Adversarial Network [39], a second model, abbreviated as Cond-SSAAE, extends the Res-SSAAE by integrating a conditional input corresponding to the activity label embedded through an embedding layer along with the sensor data. This conditional approach refines the representations of the model that could potentially lead to better separation in the latent space of the different activity. Unlike the first model, this conditional method does not function as a predictive model but rather as a cluster analysis model, since the activity performed needs to be known beforehand during both training and inference.

### 2.6. Training, Validation and Test Dataset

A user-independent k-fold cross-validation was performed with K = 4 folds, where each fold included 12 subjects for training and 4 subjects for validation (both from Group A). During training, the Kullback–Leibler (KL) divergence of the latent space for each activity is computed relative to a prior Gaussian distribution. The mean of the KL divergence values across the four activities is then calculated. The model weights are saved at the point corresponding to the lowest mean KL divergence, ensuring optimal representation of the latent space throughout the training process. This procedure stops the training when the KL value of the validation set begins to increase, preventing overfitting. During training, a learning rate of 0.0001 and the Adam optimizer [40] are used to update the model parameters. Multiple models were trained using this method to optimize the hyperparameters of the models. Subsequently, the same cross-validation was applied with the selected hyperparameters.

The model was then fine-tuned using the data from Group B for 10 epochs with a learning rate 10 times lower than the base one. Finally, the set composed of the 10 participants in Group B was used for latent space analysis. This methodology is used for analytical purposes. By initially training the base model on Group A, we enable the model to capture essential movement features. Fine-tuning the model with the data from Group B allows it to adapt to the specific characteristics exhibited during fatigue, which may be outside the distribution of Group A, ensuring that the model effectively reflects the nuances of fatigue in physical activity. Finally, using the entirety of Group B data for testing provides a comprehensive evaluation of the model’s behavior, facilitating deeper insights into how fatigue impacts performance and movement patterns. In general, this methodology supports our goal of understanding the effects of fatigue rather than focusing only on the predictive methodology.

### 2.7. Hyperparameters

The architecture of the AAE consists of three main components: the encoder, the decoder, and the discriminator and was inspired by previous research on AAEs for HAR [33,34]. The encoder model consists of three residual convolutional layers with 16, 32, and 64 output channels, respectively. Each residual layer contains a set of two convolutional layers with the ReLU activation function [41], a kernel size of 5 × 1, without pooling, and a skip connection between the input and the output of the layer without convolution. A max-pooling operation of kernel size 2 × 1 is performed between each residual block. The output of the last residual block is then connected to a dense layer with 128 neurons with the ReLU activation function. Finally, a linear layer composed of 2 neurons is added to create the latent space. The decoder is designed to be symmetric with the encoder. The models incorporate a dropout rate of 0.1 and apply batch normalization on all layers to improve training stability and performance. The discriminator consists of two dense layers, each with 512 neurons with the sigmoid activation function.

### 2.8. Cluster Evaluation

To assess the effectiveness of our fatigue monitoring approach, we evaluate the clustering of data points corresponding to each set of repetitions performed for each activity independently. Each set of 10 repetitions will form a cluster of 10 2D points. By examining and comparing these clusters, we aim to understand how fatigue influences the distribution and organization of data points within the latent space through several indices.

The Silhouette score [42] evaluates the performance of the clustering by measuring how similar an object is to its own cluster compared to the others. This index calculates the difference between the mean intra-cluster distance and the mean distance to the nearest cluster for each point [43]. A score close to 1 indicates well-separated clusters, while values near 0 suggest overlapping clusters.

The Davies–Bouldin index [44] quantifies the quality of the clustering by measuring the ratio of inter-cluster scatter to inter-cluster separation. For each cluster, the maximum similarity to any other cluster is retained, and the DB index is the average of these maximum similarities across all clusters. Lower DB values indicate better clustering, as the clusters are more compact and better separated.

In addition, the area of the confidence ellipse is used to evaluate the surface of each cluster. The confidence ellipse captures the region within which a certain percentage of data points exists [45], assuming a multivariate normal distribution that contains 95% of the data points. The confidence area ellipse helps to identify if there are any significant changes in the surface area of the clusters over time. The Euclidean distance between the centroids’ clusters is also evaluated.

Using all of the confidence area ellipse, the cluster indices, and the distance, we can comprehensively analyze the clusters: the ellipse will offer insights into the surface changes within each cluster, while the cluster index allows us to compare the current cluster (i.e., *i*th cluster) with the initial cluster (i.e., cluster 0) to detect any significant shifts or variations in the latent space. This dual approach ensures a thorough evaluation of how fatigue impacts the clustering of our data.

### 2.9. Linear Regression

Linear regression analysis is performed on all previously mentioned indices by analyzing their value across the sets. Before performing the regression analysis, it is necessary to standardize these indices for each participant separately. This standardization ensures that the indices, which may have different value ranges, are standardized to a common scale between the participants.

In addition, it is important to consider the different number of sets performed by each participant. For example, if one participant completes 50 sets, while another completes only 10, the total number of sets does not provide a consistent basis for analysis. To address this, the data are normalized by expressing the number of sets as a percentage of completion. By standardizing the indices and normalizing the number of sets, accurate assessments can be made regarding how changes in fatigue impact clustering metrics across participants.

A linear regression will be performed to model the relationships between variables and reveal trends across sets to assess the statistical significance of the change in index value (independent variable) on the percentage of completion (dependent variable). For regression analysis, an α level of 0.05 is considered a threshold for statistical significance.

## 3. Results

For each database, we performed cross-validation k=4, resulting in four distinct models for each type of model (Res-SSAAE and Cond-SSAAE). Given that there are 4 models, 4 physical activities and 4 indices, a total of 64 linear regressions are performed per database (4 models × 4 activities × 4 indices). The number of significant *p*-values (*p* < 0.05) across all four models for each type of model is then reported as the ’*p*-value counter’. This provides a summary of the significant results found for each database, separately for Res-SSAAE and Cond-SSAAE. These results are presented in Table 2 in the column “*p*-value K”.

Additionally, the column of the “*p*-value ensemble” display the mean value for each index, calculated by averaging the index values in all k=4 models for each database, effectively treating them as an ensemble to capture a more generalized result across all the models. Given that there are 4 activities and 4 averaged indices, a total of 16 linear regressions are performed per database.

**Figure 1:**
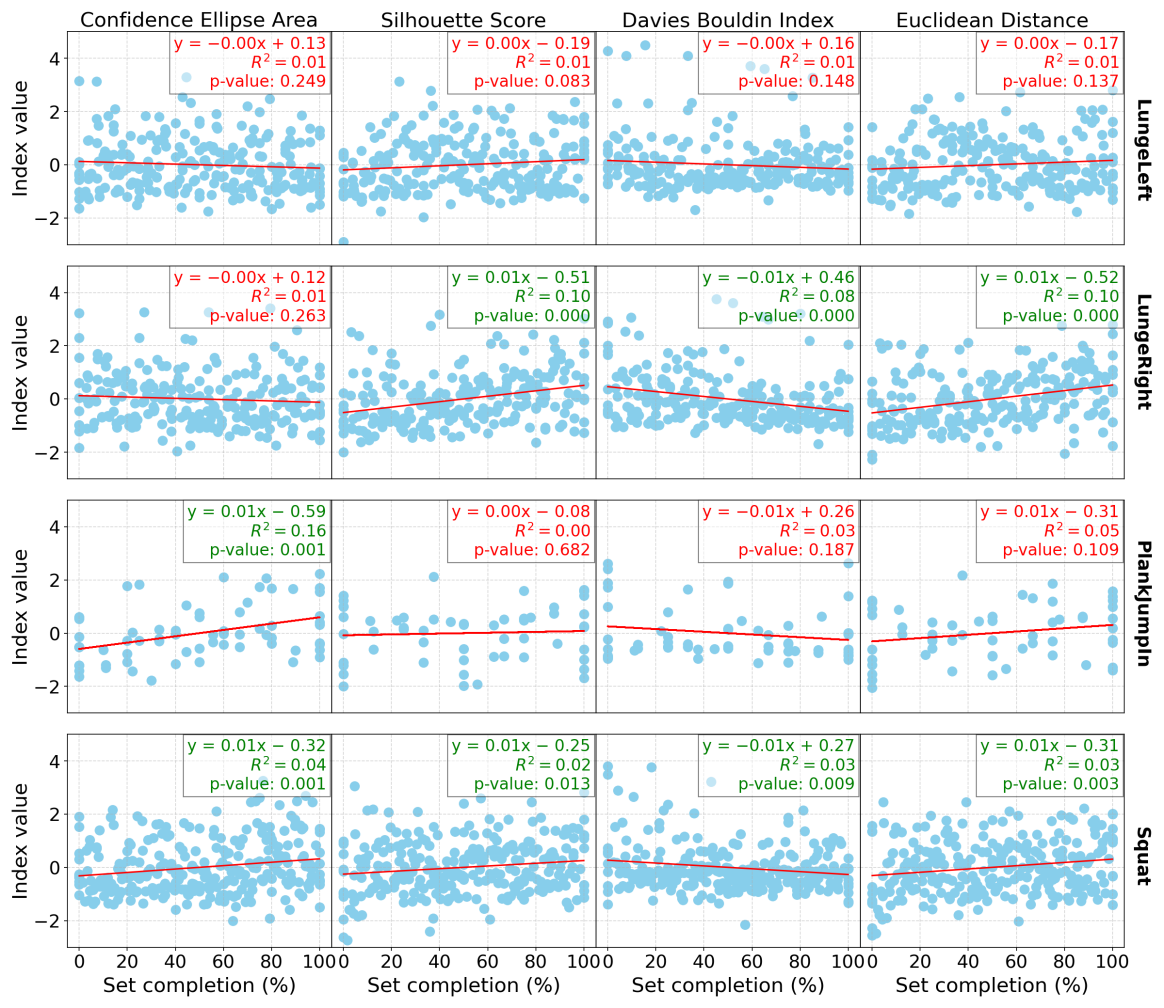
Linear regression outcomes for computed indices by exercise, with each column representing an index and each row representing a different exercise for DB0 (WiiBB). Each graph displays the linear coefficient, R², and *p*-value for the corresponding regression, with values in green indicating statistical significance (*p* < 0.05) and values in red indicating non-significance.

**Figure 2:**
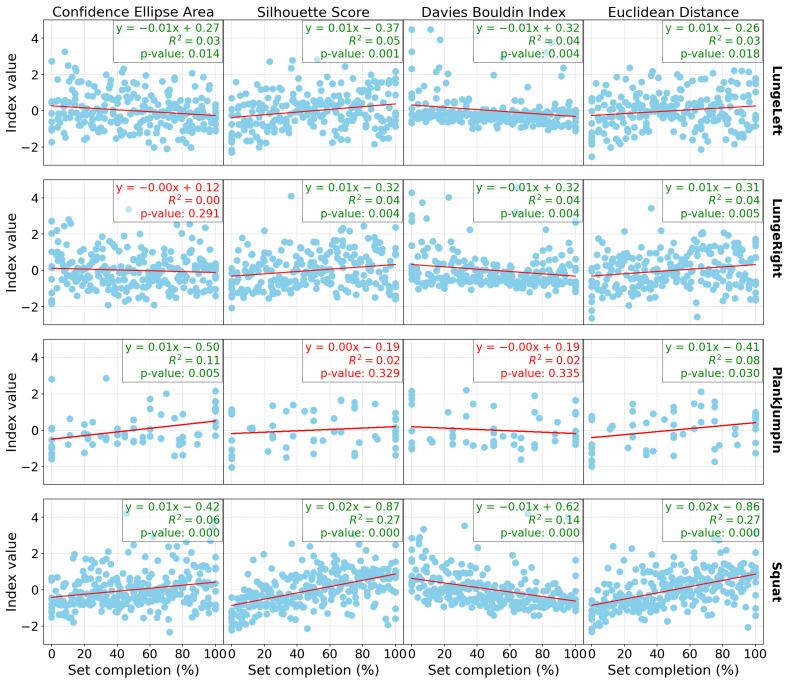
Linear regression outcomes for computed indices by exercise, with each column representing an index and each row representing a different exercise for DB3 (1 IMU on the hip). Each graph displays the linear coefficient, R², and *p*-value for the corresponding regression, with values in green indicating statistical significance (*p* < 0.05) and values in red indicating non-significance.

**Figure 3:**
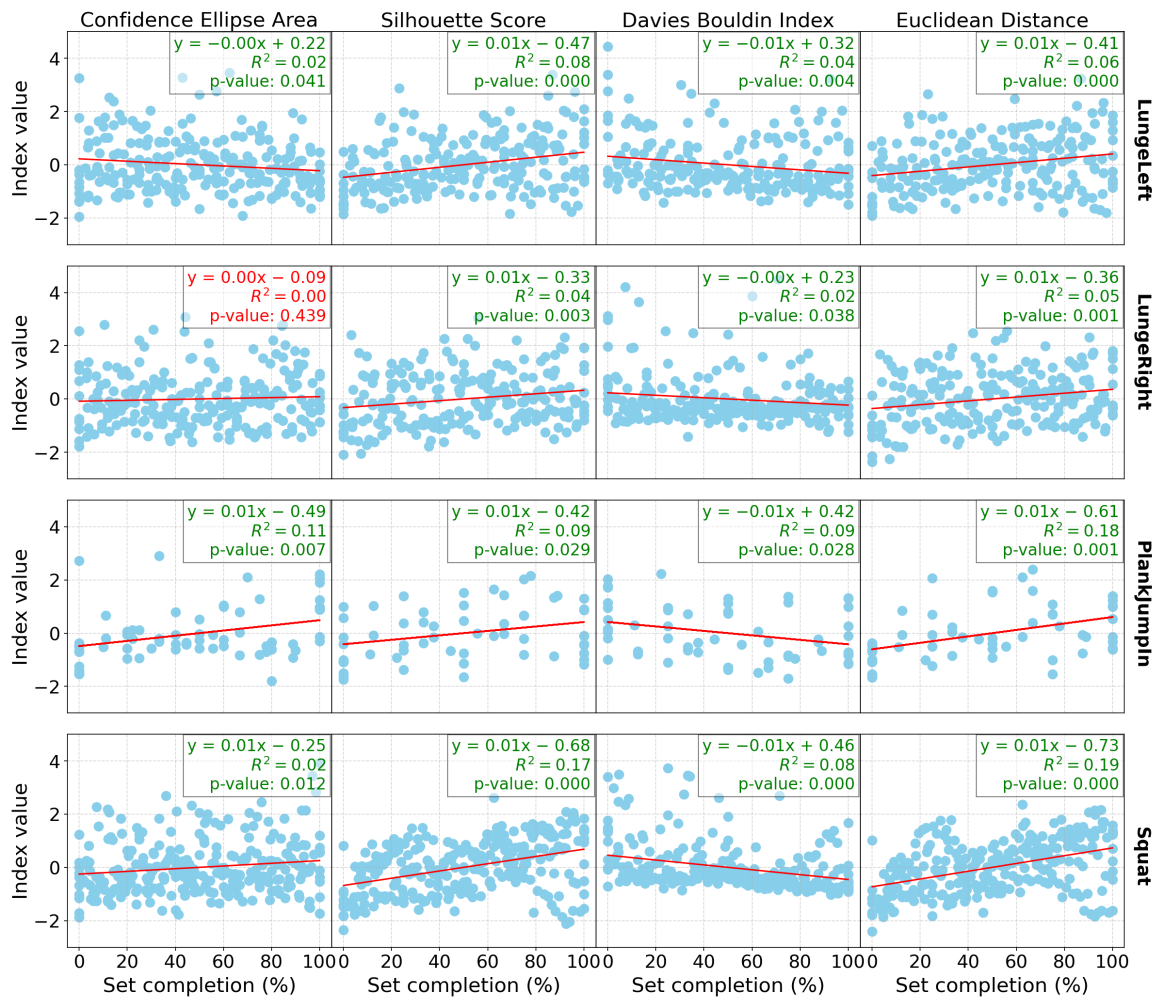
Linear regression outcomes for computed indices by exercise, with each column representing an index and each row representing a different exercise for DB4 (3 IMUs on the hip and forearms). Each graph displays the linear coefficient, R², and *p*-value for the corresponding regression, with values in green indicating statistical significance (*p* < 0.05) and values in red indicating non-significance.

Figure 1 presents the linear regression results for all the indices computed for the group of participants, as well as for each exercise individually (with one exercise per row) for the Res-SSAAE models and DB0. Similarly, Figure 2 and Figure 3 show the same linear regression results, but for DB3 and DB4, respectively. Each plot includes not only the *p*-values but also the R² values, which indicate the proportion of variance explained by the model, and the regression coefficients.

**Figure 4:**
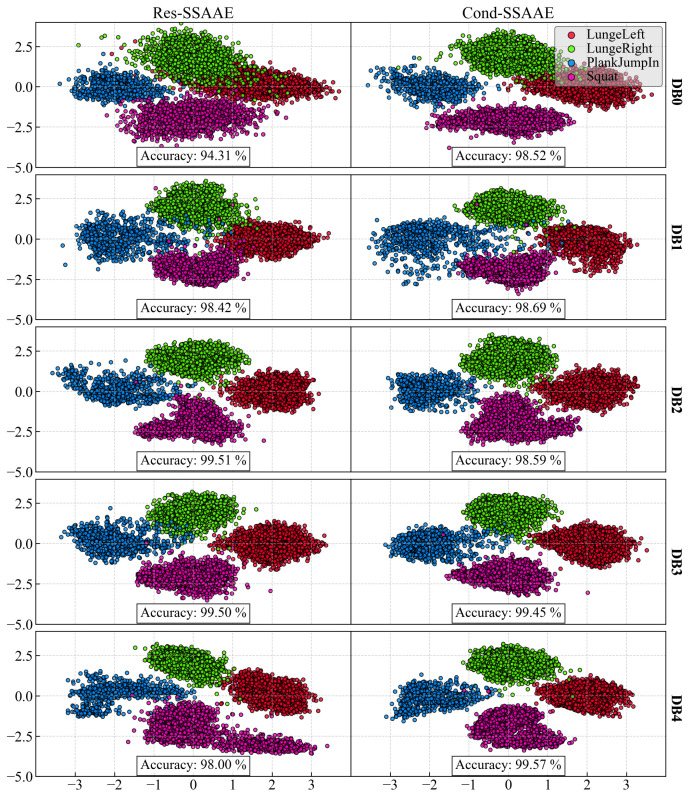
Latent space representations for Res-SSAAE (**right column**) and Cond-SSAAE (**left column**) across all databases. Each row represents a different database. The points are colored green, red, blue, and purple to represent LungeRight, LungeLeft, Plank Jump-in, and Squat, respectively. The corresponding accuracy for each latent space is displayed in the corresponding graph.

Figure 4 presents the latent space obtained for each database and model for K = 0. The latent space accuracy on the test set for Res-SSAAE is 94.31, 98,42, 99.51, 99.50, and 98.00% for DB0, DB1, DB2, DB3, and DB4, respectively. For Cond-SSAAE, the latent space accuracy is 98.52, 98.69, 98.59, 99.45, and 99.57% for DB0, DB1, DB2, DB3, and DB4, respectively.

## 4. Discussion

The purpose of this study was to use WiiBB and IMU data to evaluate the use of data dimensionality reduction models called AAEs to propose alternative classification approaches for fatigue detection [24]. The models were trained in semi-supervised and conditional settings. The encoder generated a 2D latent space and the evolution of clusters corresponding to sets of 10 repetitions in different sets of exercises performed by participants until exhaustion were analyzed. Finally, this study investigated various combinations of sensor data (WiiBB and/or IMUs) to assess their respective performance.

In terms of the database results (Table 2), both the Res-SSAAE and Cond-SSAAE models show a general upward trend in the *p*-value counter (which represents the number of significant linear regressions) as the number of sensors increases. For example, the lowest “*p*-value ensemble” counter is observed for DB0, which consists of only the Wii Balance Board (WiiBB), with 9 of 16 computed indices being significant for both models. For example, with the Res-SSAAE model, this value increases to 11 for DB1 (WiiBB + 1 IMU on the hip), 11 for DB2 (WiiBB + 3 IMUs on the hip and forearms), 13 for DB3 (1 IMU on the hip), and 15 for DB4 (3 IMUs on the hip and forearms), respectively. These results demonstrate that the use of IMUs, particularly when combined with the Wii Balance Board, is more effective than using the Wii Balance Board alone in detecting shifts in the latent space associated with fatigue. Furthermore, a clearer trend in linear regression performance is observed with DB1 and DB2 compared to DB0, as shown in Figure 1, Figure 2 and Figure 3, representing DB0, DB1, and DB2, respectively. This improvement in performance is further enhanced when multiple IMUs are combined with the Wii Balance Board.

In the latent space, it is observed that the Right and Left Lunges are poorly separated, as shown in Figure 4 for DB0 (Wii Balance Board). When activity cannot be clearly differentiated in the latent space, the model may struggle to make further distinctions, limiting its ability to identify fatigue-related patterns properly. This is evidenced in the corresponding “*p*-value counter” and “*p*-value ensemble” in Table 2. Additionally, the Plank Jump-in activity is also difficult to analyze effectively using only the Wii Balance Board. However, when three IMUs are used, the analysis improves across all activities. These results could be associated with a limitation of the Wii Balance Board, which is its low sampling rate (30 Hz), that is potentially insufficient for capturing fast, dynamic movements like Plank Jump-ins. Moreover, Wii Balance Board center-of-pressure velocity measurements have a proportional error that increases with speed [46], further limiting its accuracy for high-velocity tasks.

In Table 2 (*p*-value ensemble), Res-SSAAE shows a higher *p*-value counter for DB2, DB3, and DB4, suggesting that Cond-SSAAE may not be the preferred option when a model ensemble is available. In contrast, Cond-SSAAE appears to be the preferred choice for DB0 and DB1. This can be explained by the addition of the conditional label as an input, which allows the model to better separate the activity clusters in the latent space when fewer sensors are used. This improved separation leads to higher accuracy, as confirmed by the reported results. For example, for DB0, Res-SSAAE and Cond-SSAAE achieve accuracies of 94.31% and 98.52%, respectively.

In addition, each significant linear regression observed is associated with indices that evolve in the expected direction as fatigue increases, as shown in the latent space. For example, the area of the ellipse tends to increase, indicating greater variability in the data as fatigue progresses, which is reflected in the latent space by a wider dispersion of data points. The Silhouette score increases, reflecting better separation between clusters, while the Davies–Bouldin index decreases, confirming tighter and more distinct clusters. Additionally, the Euclidean distance between the clusters increases, further validating the separation. These trends confirm that significant changes in these indices consistently point to a shift in motor control patterns associated with fatigue observed in the latent space.

Despite the statistical significance of the linear regression results, the relatively low R² values presented in Figure 1, Figure 2 and Figure 3 suggest that the relationship between fatigue and the cluster index may not be as robust as initially perceived. This indicates that while the indices are correlated with the percentage of completion until fatigue, other underlying variables likely influence these results. One possible factor is the presence of confounding variables. Specifically, differentiating between mental and physical fatigue could be crucial, as these types of fatigue may affect motor control in different ways. The current model may not fully capture this distinction, which could explain the weaker correlations between fatigue and the indices. Another potential issue lies in the presence of noise within the latent space, which may also contribute to the low R² values. Noise can arise from various sources, including measurement errors, sensor variability, and the inherent variability in human motor control. Furthermore, the relatively small sample size in this study may limit the model’s ability to generalize, reducing statistical power, and potentially resulting in a less stable representation of the latent space. These factors may obscure the true relationship between fatigue and motor control and affect the performance of the model. Finally, the model itself may not fully capture the complexity of fatigue dynamics and motor control. The relationship between fatigue and indices may be more complex and more sophisticated modeling techniques and more diverse datasets may be needed to better capture the underlying patterns and improve the model’s ability to accurately reflect the variability in the data.

One limitation of this study is the Gaussian assumption imposed on each cluster in the latent space, which may constrain the model’s ability to fully capture the variability in the data. The Gaussian distribution forces each cluster to assume fixed shapes and sizes, limiting the flexibility of the model, particularly when the data deviate from a normal distribution, such as in the presence of outliers. This constraint may particularly limit the detection of more complex or non-linear relationships, thus restricting the ability of the model to capture the full spectrum of variability associated with fatigue. Future work should explore alternative distributions to better accommodate outliers and more accurately represent the true variability within the latent space. Non-Gaussian distribution-based approaches could provide a more nuanced representation of the data, improving the model’s ability to detect fatigue and other dynamic changes that may not align with a Gaussian distribution.

Although the model has shown some effectiveness in analyzing fatigue-related activity, further development is needed to assess its ability to generalize to new participants. The current dataset is limited in size, which may restrict the model’s ability to capture the full range of variability in fatigue during human activity. This small sample size could also contribute to the relatively low R² value observed, as it struggles to account for the diversity of fatigue patterns between participants. A larger dataset would help create a more robust latent space, allowing the model to better distinguish fatigue patterns and capture more subtle relationships in the data. This would improve the predictive capability and generalization of the model, leading to a higher R² value. Moreover, expanding the participant population to include more diverse groups (such as women and individuals with varying levels of sports experience) could further enhance the model’s capability and broaden its applicability. Furthermore, data augmentation techniques, such as time-series warping or jittering, could be applied to artificially expand the dataset, helping the model generalize better. It is important to note that both models were fine-tuned on the entire dataset with a primary focus on cluster analysis rather than prediction. It is also essential to note that the semi-supervised model can be used for predictive tasks, while the conditional model, which requires labels as input, is limited to the analysis of known activity. Future works would involve transitioning from an analysis framework to a predictive one, allowing it to effectively classify and predict outcomes for unseen data to ensure robustness and reliability in real-world applications.

## 5. Conclusions

This study used data from Wii Balance Boards and Inertial Measurement Units (IMUs) to compare the effectiveness of different sensor combinations in capturing fatigue-related changes. The objective was to explore the use of Adversarial AutoEncoders (AAEs) for dimensionality reduction to assess and visualize the impact of fatigue in a two-dimensional latent space. The focus was on understanding how fatigue affects motor control by analyzing changes in the distribution of data points within the latent space. The results demonstrated that both the semi-supervised and conditional AAEs effectively regularized the latent space by clustering each activity. However, the linear regression performance of the indices was found to be weak, suggesting the need for further optimization. Additionally, the small sample size and the lack of diversity in the population may have limited the model’s ability to generalize.

To address these limitations, future work should explore alternative cluster distributions, incorporate data augmentation, evaluate additional metrics, and use larger and more diverse datasets. Implementing data augmentation and experimenting with different latent space priors could further enhance the ability of the model to capture complex data structures and improve robustness. These improvements would enable the model to better detect subtle variations in motor control and fatigue, making it more applicable to real-world scenarios.

In general, this study highlights both the potential and challenges of using AAEs in Human Activity Recognition (HAR) to visualize and assess motor control variations associated with fatigue. This approach not only provides information on the dynamics of fatigue, but also lays the foundation for future work in training optimization and performance enhancement, with potential applications in sports and rehabilitation for personalized feedback.

## Figures and Tables

**Table 1:** List of databases considered in this study.

Database	Description
DB0	WiiBB
DB1	WiiBB + 1 IMU (hip)
DB2	WiiBB + 3 IMUs (hip + forearms)
DB3	1 IMU (hip)
DB4	3 IMUs (hip + forearms)

**Table 2:** Assessment table.

DATABASE	Model Type	*p*-Value Counter	*p*-Value Ensemble
DB0	Res-SSAAE	32/64	9/16
	Cond-SSAAE	34/64	9/16
DB1	Res-SSAAE	41/64	11/16
	Cond-SSAAE	44/64	12/16
DB2	Res-SSAAE	42/64	11/16
	Cond-SSAAE	42/64	11/16
DB3	Res-SSAAE	43/64	13/16
	Cond-SSAAE	43/64	12/16
DB4	Res-SSAAE	44/64	15/16
	Cond-SSAAE	44/64	13/16

## Data Availability

The datasets presented in this article are not readily available because they are part of an ongoing study. Requests for access to datasets for academic purposes should be directed to the corresponding authors.

## Abbreviations

The following abbreviations are used in this manuscript:

GRF	Ground Reaction Force
CoP	Center of Pressure
IMU	Inertial Measurement Unit
WiiBB	Wii Balance Board
HAR	Human Activity Recognition
AE	AutoEncoder
AAE	Adversarial AutoEncoder
SSAAE	Semi-Supervised Adversarial AutoEncoder
KL	Kullback–Leibler

## References

[1] Enoka R.M., Duchateau J. (2016). Translating Fatigue to Human Performance. Med. Sci. Sport. Exerc..

[2] Theofilidis G., Bogdanis G.C., Koutedakis Y., Karatzaferi C. (2018). Monitoring Exercise-Induced Muscle Fatigue and Adaptations: Making Sense of Popular or Emerging Indices and Biomarkers. Sports.

[3] Wan J.J., Qin Z., Wang P.y., Sun Y., Liu X. (2017). Muscle fatigue: General understanding and treatment. Exp. Mol. Med..

[4] Adão Martins N.R., Annaheim S., Spengler C.M., Rossi R.M. (2021). Fatigue Monitoring Through Wearables: A State-of-the-Art Review. Front. Physiol..

[5] Kellmann M., Kallus K.W. (2001). Recovery-Stress Questionnaire for Athletes: User Manual.

[6] McNair D.M., Lorr M., Droppleman L.F. (1992). Revised Manual for the Profile of Mood States.

[7] Alba-Jiménez C., Moreno-Doutres D., Peña J. (2022). Trends Assessing Neuromuscular Fatigue in Team Sports: A Narrative Review. Sports.

[8] Borg G.A. (1982). Psychophysical bases of perceived exertion. Med. Sci. Sport. Exerc..

[9] Morgan W.P. (1994). Psychological components of effort sense. Med. Sci. Sport. Exerc..

[10] Özgören N., Arıtan S. (2022). Peak counting in surface electromyography signals for quantification of muscle fatigue during dynamic contractions. Med Eng. Phys..

[11] Chen B., Liu P., Xiao F., Liu Z., Wang Y. (2021). Review of the Upright Balance Assessment Based on the Force Plate. Int. J. Environ. Res. Public Health.

[12] Pedley J.S., Lloyd R.S., Read P.J., Moore I.S., De Ste Croix M., Myer G.D., Oliver J.L. (2020). Utility of Kinetic and Kinematic Jumping and Landing Variables as Predictors of Injury Risk: A Systematic Review. J. Sci. Sport Exerc..

[13] Merrigan J.J., Stone J.D., Martin J.R., Hornsby W.G., Galster S.M., Hagen J.A. (2021). Applying Force Plate Technology to Inform Human Performance Programming in Tactical Populations. Appl. Sci..

[14] Lu Y., Wang J., Ren Y., Ren J. (2024). Effects of Fatigue on Ankle Flexor Activity and Ground Reaction Forces in Elite Table Tennis Players. Sensors.

[15] Liu Z., Yang C., Yu J., Zhao X., Wu J., Zhang Y., Li J., Gu Y. (2023). The Effect of Muscles Fatigue on the Knee’s Kinetics and Kinematics Characteristics. Sustainability.

[16] Kazemi Z., Mazloumi A., Arjmand N., Keihani A., Karimi Z., Ghasemi M.S., Kordi R. (2022). A Comprehensive Evaluation of Spine Kinematics, Kinetics, and Trunk Muscle Activities During Fatigue-Induced Repetitive Lifting. Hum. Factors J. Hum. Factors Ergon. Soc..

[17] Clark R.A., Mentiplay B.F., Pua Y.H., Bower K.J. (2018). Reliability and validity of the Wii Balance Board for assessment of standing balance: A systematic review. Gait Posture.

[18] Montoro-Cárdenas D., Cortés-Pérez I., Zagalaz-Anula N., Osuna-Pérez M.C., Obrero-Gaitán E., Lomas-Vega R. (2021). Nintendo Wii Balance Board therapy for postural control in children with cerebral palsy: A systematic review and meta-analysis. Dev. Med. Child Neurol..

[19] Afridi A., Rathore F.A., Nazir S.N.B. (2021). Wii Fit for Balance Training in Elderly: A Systematic Review. J. Coll. Physicians Surg. Pak..

[20] Fullerton E., Heller B., Munoz-Organero M. (2017). Recognizing Human Activity in Free-Living Using Multiple Body-Worn Accelerometers. IEEE Sensors J..

[21] Teran-Pineda D., Thurnhofer-Hemsi K., Domínguez E. (2023). Human Gait Activity Recognition Using Multimodal Sensors. Int. J. Neural Syst..

[22] Bennasar M., Price B.A., Gooch D., Bandara A.K., Nuseibeh B. (2022). Significant Features for Human Activity Recognition Using Tri-Axial Accelerometers. Sensors.

[23] Elshafei M., Shihab E. (2021). Towards Detecting Biceps Muscle Fatigue in Gym Activity Using Wearables. Sensors.

[24] Jiang Y., Hernandez V., Venture G., Kulić D., Chen B.K. (2021). A Data-Driven Approach to Predict Fatigue in Exercise Based on Motion Data from Wearable Sensors or Force Plate. Sensors.

[25] Yang J., Lee J., Choi J. (2011). Activity Recognition Based on RFID Object Usage for Smart Mobile Devices. J. Comput. Sci. Technol..

[26] Twomey N., Diethe T., Fafoutis X., Elsts A., McConville R., Flach P., Craddock I. (2018). A Comprehensive Study of Activity Recognition Using Accelerometers. Informatics.

[27] Ordóñez F.J., Roggen D. (2016). Deep Convolutional and LSTM Recurrent Neural Networks for Multimodal Wearable Activity Recognition. Sensors.

[28] Wang J., Sun S., Sun Y. (2021). A Muscle Fatigue Classification Model Based on LSTM and Improved Wavelet Packet Threshold. Sensors.

[29] Stewart T., Narayanan A., Hedayatrad L., Neville J., Mackay L., Duncan S. (2018). A Dual-Accelerometer System for Classifying Physical Activity in Children and Adults. Med. Sci. Sport. Exerc..

[30] Card S., Mackinlay J., Shneiderman B. (1999). Readings in Information Visualization: Using Vision to Think.

[31] Friendly M. (2008). The Golden Age of Statistical Graphics. Stat. Sci..

[32] Ward M., Grinstein G.G., Keim D. (2010). Interactive Data Visualization: Foundations, Techniques, and Applications.

[33] Hernandez V., Kulić D., Venture G. (2020). Adversarial autoencoder for visualization and classification of human activity: Application to a low-cost commercial force plate. J. Biomech..

[34] Kamikokuryo K., Haga T., Venture G., Hernandez V. (2022). Adversarial Autoencoder and Multi-Armed Bandit for Dynamic Difficulty Adjustment in Immersive Virtual Reality for Rehabilitation: Application to Hand Movement. Sensors.

[35] Jaramillo I.E., Chola C., Jeong J.G., Oh J.H., Jung H., Lee J.H., Lee W.H., Kim T.S. (2023). Human Activity Prediction Based on Forecasted IMU Activity Signals by Sequence-to-Sequence Deep Neural Networks. Sensors.

[36] Makhzani A., Shlens J., Jaitly N., Goodfellow I. (2015). Adversarial Autoencoders. arXiv.

[37] Goodfellow I.J., Pouget-Abadie J., Mirza M., Xu B., Warde-Farley D., Ozair S., Courville A., Bengio Y. (2014). Generative Adversarial Networks. arXiv.

[38] He K., Zhang X., Ren S., Sun J. (2015). Deep Residual Learning for Image Recognition. arXiv.

[39] Mirza M., Osindero S. (2014). Conditional Generative Adversarial Nets. arXiv.

[40] Kingma D.P., Ba J. (2014). Adam: A method for stochastic optimization. arXiv.

[41] Nair V., Hinton G.E. Rectified linear units improve restricted boltzmann machines. Proceedings of the 27th International Conference on Machine Learning (ICML-10).

[42] Rousseeuw P.J. (1987). Silhouettes: A graphical aid to the interpretation and validation of cluster analysis. J. Comput. Appl. Math..

[43] Liu Y., Li Z., Xiong H., Gao X., Wu J. (2010). Understanding of Internal Clustering Validation Measures. Proceedings of the 2010 IEEE International Conference on Data Mining.

[44] Davies D.L., Bouldin D.W. (1979). A Cluster Separation Measure. IEEE Trans. Pattern Anal. Mach. Intell..

[45] Schubert P., Kirchner M. (2014). Ellipse area calculations and their applicability in posturography. Gait Posture.

[46] Mengarelli A., Cardarelli S., Strazza A., Di Nardo F., Fioretti S., Verdini F. (2018). Validity of the nintendo wii balance board for the assessment of balance measures in the functional reach test. IEEE Trans. Neural Syst. Rehabil. Eng..

